# A role for nNOS in mediating stress and female sexual behavior in mice

**DOI:** 10.1038/s41467-024-47993-z

**Published:** 2024-04-30

**Authors:** Konstantina Chachlaki

**Affiliations:** 1 Laboratory of Development and Plasticity of the Neuroendocrine Brain, Lille Neuroscience and Cognition, Université de Lille, CHU Lille, Inserm, UMR-S 1172, F-59000 Lille, France; 2 FHU 1,000 days for Health, School of Medicine, F-59000 Lille, France; 3 University Research Institute of Child Health and Precision Medicine, National and Kapodistrian University of Athens, “Aghia Sophia” Children’s Hospital, Athens, Greece

**Keywords:** Sexual behaviour, Neurophysiology

## Abstract

Developmental stress can detrimentally affect adult female reproductive behavior, influencing sexual receptivity and fertility. Recent work has demonstrated neuronal nitric oxide (NO) synthase (nNOS)-promoted NO release in the ventromedial hypothalamus as a nexus between pre-pubertal stress and adult sexual behavior in mice.

Stress is defined as a multifaceted physiological and psychological response to challenges or demands that threaten psychosomatic homeostasis. Stress has been linked to negative health outcomes through its biological impact on autonomic and neuroendocrine responses, affecting both the hypothalamic-hypophyseal-adrenocortical (HPA) and hypothalamic-hypophyseal-gonadal (HPG) systems. Indeed, stress is a profound disruptor of both female and male fertility, compromising various aspects of reproductive function, such as libido, spermatogenesis, and ovulatory capacity, while also affecting the release of gonadal hormones, potentially prioritizing individual survival over species maintenance^1^. Recently, numerous studies have suggested that alterations in stress hormones during vulnerable periods of development, such as early life, childhood, and adolescence, can have serious health consequences^2^. Early-life adversity may leave behind an epigenetic imprint, persisting into adulthood and old age, suggesting a distinctive pattern of gene expression, potentially affecting the susceptibility to stress^3^.

In a recent study, Bakker and colleagues demonstrate that in mice social-isolation-induced stress during the critical developmental period preceding pubertal onset, i.e., between postnatal days 21–50, leads to persistent changes in the reproductive behavior, as demonstrated by the decrease in sexual receptivity and the number of ovulatory events of female adult mice. (Stress during pubertal development affects female sociosexual behavior in mice.) These long-lasting alterations in female sexual behavior align with the notion that puberty is a period vulnerable to stress disruptions in the maturation of neural circuits governing reproductive physiology. Notably, the authors identify two subpopulations within the stressed female group: the minimally sexually receptive (MR) and highly receptive (HR) females. MR females exhibit a lower lordosis quotient, a characteristic posture during mating, and a higher rejection quotient, indicating reduced sexual receptiveness, while HR females do not significantly differ from the non-stressed control subjects (Fig. 1A). Although puberty was not directly assessed, the variations between MR and HR females may reflect differences in the individual capacity to develop homeostatic adaptive mechanisms, potentially prepubertally. This raises the intriguing question of whether stress directly affects sexual behavior or indirectly influences it through potential disruptions in the timing of puberty, known to impact sexual function.

**Fig. 1:**
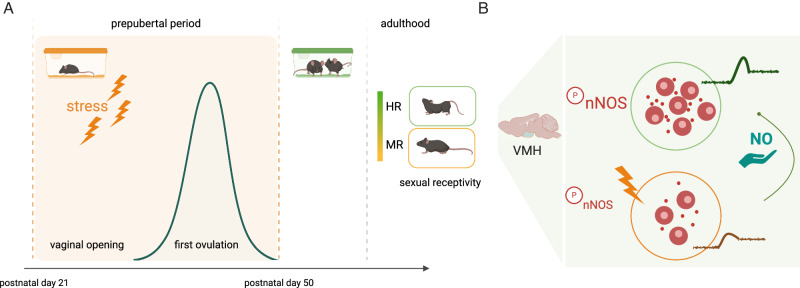
Impact of peripubertal stress on female sexual behavior: the involvement of the nNOS population in the ventromedial hypothalamic area. Stress during the critical developmental period preceding puberty induces enduring alterations in neural circuits and activity (**A**), specifically in the ventromedial hypothalamus (VMHvl), mediated by the neurons expressing the neuronal nitric oxide synthase (nNOS) (**B**). The intricate interplay of stress, sex hormones, and the nNOS pathway sheds light on the complex mechanisms influencing reproductive behavior in adulthood. This figure was produced using BioRender.

Chronic stress, especially during critical developmental periods such as puberty, is believed to disrupt the adaptive dynamics of the HPA axis, leading to persistent sensitization of the stress response. This includes increased production of corticotropin-releasing hormone (CRH), triggering adrenocorticotropin hormone (ACTH) secretion from the pituitary gland, and subsequently promoting the secretion of glucocorticoids and adrenal androgens. Elevated stress hormone levels can suppress the HPG axis, impacting neural circuits involved in sexual behavior and contributing to variations in sexual receptivity and related behaviors. To investigate the involvement of HPA dysregulation in the altered sexual behavior in peripubertally-stressed females, the researchers employed the “match-mismatch” model^2^, suggesting that early-life changes in HPA regulation may result in inappropriate responses to stressors encountered later in life. Analysis of corticosterone and DHEA levels following additional adult exposure to stress revealed no difference in the baseline or adult stress-response corticosterone/DHEA ratio between the peripubertally-stressed and control groups. Interestingly, the reduced sexual performance of the peripubertally-stressed animals did not correlate with anxiety or depression-like behaviors in adulthood, suggesting that the aberrant sexual behavior is not an inevitable, indirect result of profound HPA axis dysfunction. In addition, contrary to the expected effects of chronic stress on gonadal steroids, reduced sexual performance did not correlate with changes in the circulating levels of steroid hormones peripubertal (at postnatal day 40). This finding is supported by the unaltered number of hypothalamic kisspeptin neurons, a neuronal population highly sensitive to circulating estradiol throughout postnatal development^4^, as well as the unaltered sexual preference, which is orchestrated by sex hormones. Although the GnRH/LH release pattern was not assessed, the correlation between peripubertal stress and impaired estrous cyclicity suggests a dysfunction of the reproductive axis in adulthood. Given recent studies revealing that alterations in the GnRH release pattern during development result in an aberrant rhythmic LH release in adulthood, correlating with adverse psychosomatic consequences^5,6^, including impaired sexual behavior^5^, future studies should assess whether peripubertal-stress response involves a disrupted GnRH network.

Exposure to stress by social isolation during puberty has been demonstrated to induce alterations in the distribution of estrogen receptor alpha (ERα) in key brain regions such as the preoptic area and the ventrolateral part of the ventromedial hypothalamus (VMHvl)^7^ in mice. This suggests that stress may affect female sexual function by altering the mediation of sex steroid information in the hypothalamus rather than the hormonal levels per se. Surprisingly, immunohistochemical analysis of the VMHvl indicates that peripubertal stress does not affect the total number of neurons expressing ERα or the progesterone receptor (PR) despite their significant roles in female sexual behavior^8^. However, in response to male bedding, the overall activity of neurons located in the VMHvl, assessed by Fos expression, was significantly decreased in the peripubertally-stressed females. This altered activation pattern did not occur in other hypothalamic nuclei involved in sexual behavior, such as the anteroventral periventricular zone of the preoptic area (AVPV) containing the essential for female sexual behavior kisspeptin neurons^9^, suggesting the involvement of a neural pathway acting in the VMHvl.

Previous studies have highlighted the crucial role of neuronal nitric oxide (NO) synthase (nNOS)-expressing neurons in the VMHvl (nNOS^VMHvl^) in the processing and integration of olfactory signals, such as male urinary odors, essential for the manifestation of female reproductive behaviors^10,11^. In addition, hypothalamic nNOS neurons are essential for the postnatal maturation of the HPG axis, with their activity being highly sensitive to environmental and hormonal changes^5,12^. Exploring whether alterations in the nNOS population contribute to the observed VMHvl dysfunction, including the decline in the overall VMHvl activation pattern, the authors revealed a significant reduction in the number of nNOS neurons located in the VMHvl in peripubertal-stressed adult females and a significant decrease in the Fos expression in nNOS^VMHvl^ neurons following adult exposure to male olfactory cues. (Stress during pubertal development affects female sociosexual behavior in mice.) Using fiber photometry, the results demonstrated that MR females exhibited a blunted activation of nNOS neurons in response to male urinary odors compared to both control and HR females.

Given that, in both the POA and VMHvl, the majority of nNOS neurons co-express ERα^13^, the authors assessed whether the observed dysregulation of nNOS neurons in the VMHvl was associated with a specific reduction in the co-expression of ERα. Indeed, further analysis revealed a significant reduction in the number of nNOS neurons co-expressing ERα in MR females compared to the control subjects. (Stress during pubertal development affects female sociosexual behavior in mice.) To examine whether the activation of nNOS^VMHvl^ neurons by urinary olfactory cues could be influenced by sex steroids, the authors recorded nNOS neurons, using fiber photometry, under different hormonal conditions. Activation of nNOS^VMHvl^ neurons in response to male urinary odor was attenuated in ovariectomized females, while it was highest in ovariectomized mice treated with both estradiol and progesterone. (Stress during pubertal development affects female sociosexual behavior in mice.) This underscores a synergistic effect of sex steroids on the activation response of nNOS^VMHvl^ neurons, raising additional questions about the mechanism acting upstream of the nNOS^VMHvl^ neurons following the synergistic effects of estrogen and progesterone.

Kisspeptin is a known upstream regulator of nNOS enzymatic activity, promoting the phosphorylation of nNOS enzyme at the Ser1412 activation site, leading to endogenous NO release^14^, while it has been previously shown to promote lordosis behavior acting via the nNOS neurons in the VMHvl^11^, hinting that the nNOS^VMHvl^ population might be modulating female sexual behavior under the control of kisspeptin. However, peripheral administration of kisspeptin did not affect the lordosis quotient of the MR females (Stress during pubertal development affects female sociosexual behavior in mice.), suggesting that stress-induced alterations in nNOS^VMHvl^ neuronal activity do not involve posttranslational protein modifications or do not directly affect nNOS enzymatic activity. Alternatively, it might indicate a failure of peripheral kisspeptin to reach and/or sufficiently activate the nNOS^VMHvl^ population, as observed in previous studies where kisspeptin was ineffective in promoting nNOS phosphorylation in the tuberal hypothalamus^15^. Identifying alternatives to kisspeptin gonadal-dependent mechanisms intricately modulating nNOS activity becomes an exciting research question. Intriguingly, when the authors directly manipulated NO levels by the peripheral or local administration of exogenous NO in the form of an NO donor (SNAP), they successfully restored disrupted lordosis behavior in adult MR females. The increased lordosis quotient of MR females following exogenous NO correlated with an increase in the number of phosphorylated nNOS^VMHvl^ neurons (Fig. 1B).

Intriguingly, despite MR females having fewer nNOS neurons in the VMHvl, the number of phosphorylated nNOS neurons remained unaltered between control and MR females in both vehicle and SNAP-injected conditions. Although the nNOS/phospho-nNOS ratio in MR females was not assessed, the findings of the present study suggest that the nNOS^VMHvl^ activation rate per se may be increased in peripubertally-stressed females. If so, this could potentially reflect a compensatory mechanism in response to the peripubertal stress-induced decrease in the number of nNOS^VMHvl^ neurons. Alternatively, it may suggest a direct action of nNOS-produced NO on nNOS expression rather than nNOS enzymatic activity per se. NO has been previously implicated in the transcriptional regulation of NOS expression^16^ and is recognized as an architect of epigenetic landscapes. Future work should consider the influence of genetic variations within the VMHvl, as well as the role of the epigenome, which is known to guide early developmental stages, in shaping individual susceptibility to stress-induced sexual behavior responses.

Overall, the current study presents promising avenues for future research into the potential consequences of peripubertal stress on neural plasticity and the development of adaptive neuronal circuits, especially within the VMHvl. Investigating potential interactions of the NO pathway with other systems, acting upstream or downstream of nNOS^VMHvl^ neurons, could offer insights into the intricate neurochemical regulation of sociosexual behavior. A crucial consideration is whether the observed effects can be altered or prevented. The correction of adult sexual behavior through NO therapy in prepubertally-stressed females (Stress during pubertal development affects female sociosexual behavior in mice.), combined with the recently emphasized significance of early-life NO in HPG axis maturation and adult sexual function in mice^5^, underscores the importance of investigating the mechanistic details of early-life dysfunction and their lasting impact on adult sexual behavior.
